# Evaluation of Microbiological and Chemical Contaminants in Poultry Farms

**DOI:** 10.3390/ijerph13020192

**Published:** 2016-02-04

**Authors:** Justyna Skóra, Katarzyna Matusiak, Piotr Wojewódzki, Adriana Nowak, Michael Sulyok, Anna Ligocka, Małgorzata Okrasa, Janusz Hermann, Beata Gutarowska

**Affiliations:** 1 Faculty of Biotechnology and Food Science, Institute of Fermentation Technology and Microbiology, Lodz University of Technology, 90 924 Łódź, Poland; justyna.skora@p.lodz.pl (J.S.); adriana.nowak@p.lodz.pl (A.N.); beata.gutarowska@p.lodz.pl (B.G.); 2 Department of Environmental Chemistry, University of Technology and Life Science in Bydgoszcz, 85 796 Bydgoszcz, Poland; piotr.wojewodzki@utp.edu.pl (P.W.); hermann@utp.edu.pl (J.H.); 3 Department of Agrobiotechnology, Center for Analytical Chemistry, University of Natural Resources and Life Sciences Vienna (BOKU), 3430 Tulln an der Donau, Austria; michael.sulyok@boku.ac.at; 4 Department of Microbiology and Food Technology, University of Technology and Life Science in Bydgoszcz, 85 796 Bydgoszcz, Poland; ligocka@utp.edu.pl; 5 Department of Personal Protective Equipment, Central Institute for Labour Protection, National Research Institute, 00 701 Warsaw, Poland; maokr@ciop.lodz.pl

**Keywords:** poultry farm, microorganisms, air quality, VOCs, mycotoxins, settled dust

## Abstract

The aim of the study was to evaluate the microbiological and chemical contamination in settled dust at poultry farms. The scope of research included evaluating the contributions of the various granulometric fractions in settled dust samples, assessing microbial contamination using culture methods, concentrations of secondary metabolites in dust and their cytotoxicity against hepatocyte chicken cells by means of MTT (3-(4,5-dimethylthiazolyl-2)-2,5-diphenyltetrazolium bromide) tests. In addition, we also evaluated the concentration of selected volatile odorous compounds (VOCs) using gas chromatographic and spectrophotometric methods and airborne dust concentration in the air with DustTrak™ DRX Aerosol Monitor. Studies were carried out on chicken broilers and laying hens at 13 poultry farms, with numbers of birds ranging from 8000 to 42,000. The airborne total dust concentration at poultry farms averaged 1.44 mg/m^3^ with a high percentage of the PM_10_ fraction (particulate matter with a diameter less than 10 μm). Microorganism concentrations in the settled dust were: 3.2 × 10^9^ cfu/g for bacteria and 1.2 × 10^6^ cfu/g for fungi. Potential pathogens (*Enterococcus* spp., *Escherichia coli*, *Salmonella* spp., *Aspergillus fumigatus*, *Paecilomyces variotii*) were also found. Secondary metabolites included aurofusarin, deoxynivalenol, 15-hydroxyculmorin zearalenone, zearalenone-sulfate, infectopyron, and neochinulin A. However, the dust samples showed weak cytotoxicity towards chicken hepatocyte cells, which ranged between 9.2% and 29.7%. Among volatile odorous compounds ammonia, acrolein, methyloamine, acetic acid, acetoaldehyde and formaldehyde were detected in the air. In conclusion, settled dust can be a carrier of microorganisms, odours and secondary metabolites in poultry farms, which can be harmful to workers’ health.

## 1. Introduction

Animal production can pose serious problems to both workers, animal health, and the surrounding environment. Poultry production may be connected to high concentrations of organic dust, microorganisms in manure, litter, dust and air, and the emission of volatile odorous compounds. Organic dust in poultry farms is a complex mixture of organic and inorganic particles from faecal material/manure, feed, litter, feathers, dander (skin material), mites, bacteria, fungi and fungal spores and endotoxins, depending on the type of bird and stage of the production cycle [1]. Poultry dust may contain bacteria and fungi of plant and animal origin at concentrations ranging between 10^4^ and 10^7^ cfu/g [2,3]. The presence of mould species: *Acremonium, Alternaria, Aurobasidium Aspergillus*, *Basidiospores, Cladosporium, Chrysosporium, Drechslera, Epicoccum, Eurotium, Fusarium, Geomyces, Mucor, Penicillium, Pithomyces, Rhizomucor, Scopulariopsis* and *Ulocladium* have been reported to be prevalent in poultry dust [4]. Many of them (e.g., *Alternaria, Aspergillus, Fusarium*) are recognised as allergenic strains. Directive 2000/54/EC [5] lists microorganisms in the manure of industrial poultry farms that can potentially be hazardous. Among these, Group 3 organisms include *Bacillus anthracis*, *Chlamydia ornithosis*, *Salmonella choleraesuis var.* Typhi, H5N1 virus; while Group 2 include *Aspergillus fumigatus, Candida albicans, Cryptococcus neoformans*, *Listeria monocytogenes*, *Mycoplasma* spp. *Staphylococcus* spp., *Streptococcus* spp., [4,6]. These and other pathogens may be harmful both to poultry farm workers and birds. Moreover, dust contains biological toxins, including immunotoxicity factors, for example bacterial endotoxin, fungal mycotoxins and glucans, volatile odorous compounds, plant toxins, animal venoms [7,8].

The literature suggests that people working on poultry farms are exposed to higher levels of organic dust compared to those working in cow or swine breeding [9]. Organic dust, which penetrates the respiratory system of workers, may induce toxicity, irritation, allergies, cancer or fibrosis, and result in diseases such as chronic obstructive lung disease, asthma, chronic bronchitis, bronchial hyperreactivity, organic dust toxic syndrome, and irritation of the mucous membranes of the conjunctiva and skin [10,11]. Donham *et al.* [12] reported a decline of lung function in poultry workers. Chronic respiratory symptoms, decreased lung function, rhinitis or eczema were symptoms identified among poultry workers with more than 5 years of occupational exposure [12].

Poultry farms are the biggest emitters of volatile odorous compounds. On poultry farms these are the result of fermenting and rotting litter, and also due to the decomposition of manure and debris. Part of the gas may result from breathing, digestion and release from the skin of the birds. Research is still ongoing to identify all odorous gases in livestock with current estimates identifying approximately 130 compounds. These include hydrogen sulphide, ammonia, thiols, sulphides and aliphatic amines, heterocyclic organic compounds containing sulphur and nitrogen, aliphatic alcohols and phenols, ketones, aldehydes, aliphatic acids, and esters [13]. According to the Reference Document on Best Available Techniques for Intensive Rearing of Poultry and Pigs (ILF BREF), and The European Pollutant Release and Transfer Register (E-PRTR), the main pollutants emitted from poultry houses are ammonia (NH_3_), methane (CH_4_), nitrogen monoxide (N_2_O), and inspirable and respirable dust [14,15]. Moreover, odorous emissions from poultry houses are highly variable. It depends on numerous factors including the age of the birds, the season, dietary composition, temperature, humidity, litter type and quantity, manure handling operations, bird density, and the type and rate of ventilation [16,17,18,19,20,21].

Nevertheless, legislations concerning environmental protection (Directive 2008/50/EC, 2008) and animal welfare (Council Directive 2007/43/EC, 2007) set limits on the concentration of only certain substances: carbon dioxide (3000 ppm) and ammonia (20 ppm) [22,23]. In addition, there are no established regulations on the permissible levels of settled dust in livestock, types of microorganisms and their secondary metabolites. Therefore, it is necessary to undertake additional research on the microbiological and chemical characteristics of dust present in poultry farms, which may be a health threat to workers and animals. Currently, there is limited knowledge on the cytotoxicity of settled dust in poultry farms towards chicken hepatocytes cells.

The aim of the study was to evaluate the microbiological and chemical contamination in settled dust in poultry farms. The scope of research included evaluating the contributions of various granulometric fractions in settled dust samples, assessing microbial contamination, determining the concentrations of secondary metabolites and their cytotoxicity against hepatocyte chicken cells. In addition, we also evaluated concentrations of selected odorous volatile compounds in the air of the poultry farms.

## 2. Materials and Methods

### 2.1. Poultry Farms and Dust Samples

Analysis was performed between weeks 3 and 57 of the breeding cycle (farms I-X—broilers kept on deep litter systems; farms XI-XIII—laying hens kept in caged systems) at 13 poultry farms located in Kuyavia-Pomerania and Lodz districts (Poland) containing from 8000 to 42,000 birds. Table 1 summarizes the characteristics of the livestock buildings.

**Table 1:** Livestock building descriptions.

Poultry Farm	Description	Birds
I ^B^	Area: 1831 m^2^, feeders (4 lines), 5 drip-type drinker lines, 12 roof-mounted fans, 4 wall-mounted fans	37,500
II ^B^	Area: 1260 m^2^, feeders (2 lines), 3 drip-type drinker lines, 9 roof-mounted fans, 4 wall-mounted fans	25,000
III ^B^	Area: 1750 m^2^, feeders (3 lines), 4 drip-type drinker lines, 18 roof-mounted fans, 4 wall-mounted fans	33,000
IV ^B^	Area: 2100 m^2^, feeders (4 lines), 5 drip-type drinker lines, 18 roof-mounted fans, 13 wall-mounted fans	42,000
V ^B^	Area: 1566m^2^, feeders lines (5 lines), 6 drip-type drinker lines, 11 roof-mounted fans	30,000
VI ^B^	Area: 1383m^2^, feeders (3 lines), 4 drip-type drinker lines, 16 roof-mounted fans, 2 wall-mounted fans	27,400
VII ^B^	Area. 1085m^2^, feeders (3 lines), 4 drip-type drinker line, 6 roof-mounted fans, 3 wall-mounted fans	18,000
VIII ^B^	Area: 1750 m^2^, feeders (3 lines), 4 drip-type drinker lines, 18 roof-mounted fans, 4 wall-mounted fans	33,600
IX ^B^	Area: 1831 m^2^, feeders (4 lines), 5 drip-type drinker lines, 12 roof-mounted fans, 4 wall-mounted fans	37,500
X ^B^	Area: 1074 m^2^, feeders (3 lines), 4 drip-type drinker lines, 11 wall-mounted fans	24,000
XI ^H^	Area: 1430 m^2^, feeders (3 lines), 4 drip-type drinker lines, 11 wall-mounted fans	8000
XII ^H^	Area: 1080 m^2^, feeders lines (2 lines), 4 drip-type drinker lines, 10 roof-mounted fans, 2 wall-mounted fans	8000
XIII ^H^	Area: 1200 m^2^, feeders (lines 6), 7 drip-type drinker lines, 6 roof-mounted fans, 4 wall-mounted fans	27,800

B—broilers on deep litter systems; H—laying hens in caged systems.

Settled dust is the part of airborne particulate matter that fell down onto sampling surface during the chicken broiler production cycle. Three metal plates were set at a height of about 1.6 m, evenly in both ends and in the middle of every analyzed poultry house. The sampling plates were placed in the poultry buildings the day before introducing chickens and on the last day of production cycle, after removing chickens from the building, the sampling plates containing settled dust were collected. Settled dust samples were collected after the production cycle on three sampling surfaces (metal plates; surface dimensions listed in Table 2) in each chicken broiler house. Samples were carefully swept with disposable brushes into polypropylene string bags, then mixed together and used for further analyses. The average mass (weight (g)) of dust collected is also presented in the Table 2. Microbial contamination, secondary metabolites and cytotoxicity were determined in the dust samples (farms I-X), and airborne dust concentration in farm XIII. Analyses of selected volatile odorous compounds in the air were undertaken for poultry farm II at three stages of the production cycle: stage I (without chickens); stage II (20th day), stage III (35th day), and stages XI and XII (57th week, at the end of the production cycle). The research was carried out between January and July 2015.

**Table 2:** Settled dust sample characteristics.

Poultry Farm	Collection Surface (m^2^)	Weight (g) *	Humidity (%)	Fall (g/cm^2^)
I	1.14 ± 0.00	21.32 ± 6.54	23.35 ± 8.07	0.0019 ± 0.0006
II	1.01 ± 0.00	9.81 ± 1.25	19.13 ± 3.41	0.0010 ± 0.0001
III	0.86 ± 0.00	32.63 ± 15.79	8.62 ± 0.43	0.0038 ± 0.0018
IV	0.86 ± 0.00	25.10 ± 16.34	8.85 ± 0.26	0.0029 ± 0.0019
V	0.32 ± 0.00	8.18 ± 3.57	6.52 ± 0.36	0.0031 ± 0.0023
VI	0.37 ± 0.00	13.29 ± 0.29	6.28 ± 0.47	0.0036 ± 0.0010
VII	0.33 ± 0.00	13.13 ± 0.21	8.58 ± 0.41	0.0039 ± 0.0010
VIII	0.20 ± 0.00	8.34 ± 5.08	6.32 ± 0.49	0.0042 ± 0.0026
IX	0.09 ± 0.00	5.57 ± 0.44	10.81 ± 0.31	0.0061 ± 0.0050
X	0.09 ± 0.00	4.29 ± 0.21	10.42 ± 0.54	0.0047 ± 0.0020

* average mass of dust collected on three sampling plates from each chicken broiler house.

### 2.2. Airborne Dust

Airborne dust concentration was measured using a portable laser photometer DustTrak™ DRX Aerosol Monitor 8533 (TSI, Shoreview, MN, USA), which allows simultaneous measurements of size-segregated mass fractions corresponding to PM_1_ (Particulate Matter), PM_2.5_, PM_4_ (respirable), PM_10_ and total PM size fractions. Measurements were carried out five times for 15 min with a sampling interval of 1 s (the number of samples for each measurement was *n* = 900). Dust concentration data were then used to calculate 8 h equivalent time-weighted averages, but as the measurements only lasted for short periods, the calculated values should be treated as estimates [24].

### 2.3. Size Fractions of Settled Dust

Samples of settled dust from three sampling points in each poultry farm were taken for granulometric fraction analysis (the total number of samples was 60). A laser diffraction dust particle size analyser, Mastersizer 2000 ver. 5.60, equipped with a Hydro 2000MU wet sample dispersion unit (Malvern Instruments, Ltd.; Malvern, UK), was used for various granulometric fraction analyses of the settled dust samples. The dust fraction PM_2.5_ (respirable dust with a diameterless than 2.5 μm), PM_10_ (particulate matter with a diameter less than 10 μm) and PM > 10 (particulate matter with a diameter more than 10 μm) were each analysed in triplicates and presented as the percentage of settled dust.

### 2.4. Microbial Contamination

Samples of settled dust from each poultry farm were analysed microbiologically. For this purpose, 4–32 g samples of settled dust were collected in sterile bins, mixed, and a 1 g sample was suspended in 99 mL saline solution (0.85% NaCl). The samples were diluted from 10^−1^ to 10^−8^ in duplicates and plated on MEA (Merck Millipore, Darmstadt, Germany) medium with (0.1%) chloramphenicol (fungi); Nutrient agar (Merck) with (0.2%) nystatin (bacteria); Actinomycete Isolation Agar (Hi Media Laboratories, Maharashtra, India) with (0.2%) nystatin (actinomycetes); Chapman agar (mannitol-positive *Staphylococcus* spp.), King B medium (Hi Media Laboratories) (*Pseudomonas fluorescens*), ENDO medium (Merck) (*Escherichia coli*); Kanamycin Aesculin Azide medium (Merck) (*Enterococcus* spp.); BPLA medium, following Kaufmann (Merck) (*Salmonella* spp.) Samples were incubated at 37 ± 2 °C for 24–48 h (total number of bacteria, mannitol-positive *Staphylococcus* spp., *Escherichia coli, Enterococcus* spp., *Salmonella* spp.), at 27 ± 2 °C for 5–7 days (fungi, actinomycetes), or at 30 ± 2 °C for 48 h (*Pseudomonas fluorescens*). After incubation, the colonies were counted, and the results were expressed in cfu/g of dust. Data was analysed from three independent experiments. The final result was calculated as the arithmetic mean and standard deviation (SD) of all repetitions.

The pure cultures of yeast and moulds were characterized macroscopically, and then, identified. For yeasts, diagnostics was performed using the API C AUX test (BioMérieux, Marcy-l'Étoile, France). Isolated moulds were identified by macroscopic and microscopic observations of culture on CYA (czapek yeast extract agar) and YES (yeast extract with supplements) media, using taxonomic keys [25,26,27,28]. Percentage of isolated mould colonies and the frequency of isolation (the percentage of samples in which isolated the species) for the identified species were determined.

### 2.5. Secondary Metabolites

Secondary metabolite analyses were performed for 10 settled dust samples taken from each tested poultry farm. For this purpose 4–32 g of dust were collected in sterile bins and mixed. Next, 0.5 g of dust samples were suspended in 5 mL of the extraction solvent (acetonitrile/water/acetic acid 79:20:1, v:v:v). Samples were extracted for 90 min and diluted with the same volume of solvent prior to injection [29] Secondary metabolite concentrations were analysed quantified using LC-MS/MS, as described by Sulyok *et al.* [30] with further modification. Parameters for liquid chromatography and mass spectrometry are described elsewhere [31]. Briefly, LC-MS/MS screening of target microbial metabolites was performed with a QTrap 5500 LC-MS/MS System (Applied Biosystems, Foster City, CA, USA) equipped with a TurboIonSpray electrospray ionization (ESI) source and a 1290 Series HPLC System (Agilent, Waldbronn, Germany). Chromatographic separation was performed at 25 °C on a Gemini^®^ C18-column, 150 × 4.6 mm i.d., 5 mm particle size, equipped with a C18 4 × 3 mm i.d. security guard cartridge (Phenomenex, Torrance, CA, USA). ESI-MS/MS was performed in the time-scheduled multiple reaction monitoring (MRM) mode, both in positive and negative polarities, in two separate chromatographic runs per sample, by scanning two fragmentation reactions per analyte. The MRM detection window of each analyte was set to its expected retention times of ±27 and ±48 s in the positive and negative modes, respectively. The positive analyte was confirmed by the acquisition of two MRMs per analyte, with the exception of moniliformin, which exhibited only one fragment ion. This yielded 4.0 identification points according to the European Union Commission decision 2002/657 [32]. The LC retention time and the intensity ratio of the two MRM transitions agreed with the related values of an authentic standard within 0.1 min and 30% rel. Analysis was performed in three replicates per sample.

### 2.6. Cytotoxicity

Cytotoxicity analysis was performed for 10 settled dust samples taken from each tested poultry farm. For this purpose, 4–32 g of dust were collected in sterile bins and mixed. Next, water-soluble fraction extracts were prepared: 0.1 g of dust samples were suspended in 20 mL of PBS—Phosphate Buffered Saline (Sigma-Aldrich, St. Louis, MO, USA) (dust concentration in extracts was 0.5%), pH7.2. Samples were extracted for 30 min, next 3 mL of each extract was filtered by sterile syringe filters (0.22 μm) (MerckMillipore, Darmstadt, Germany). Cytotoxicity of the prepared extracts was determined using the MTT assay with chicken liver hepatocellular carcinoma cell line, Leghorn male hepatoma (LMH) (CLS, Eppelheim, Germany, lot no. 601411-714SF), after 24 passages.

Once the cells became adherent they were cultured in collagen coated Roux flasks (BioCoat, Becton, Dickinson and Co., Franklin Lakes, NJ, USA) as a monolayer in Waymouyh’s Medium (Gibco, Thermo Fisher Scientific, Waltham, MA, USA) with the addition of 7.5% sodium bicarbonate (Gibco), 10% FBS heat-inactivated foetal bovine serum (Gibco), 25 mM HEPES (Sigma-Aldrich), 100 IU/mL penicillin (Sigma-Aldrich), and 100 μg/mL streptomycin (Sigma-Aldrich). The cells were incubated in a CO_2_ incubator at 37 °C in 5% CO_2_ for 7 days to reach 80% confluency. The medium was changed every 3–4 days. After reaching confluence, the cells were sub-cultured. They were detached with TrypLE™ Express (Gibco) for 5 min at 37 °C, suspended in sterile PBS and aspirated off the plastic flask. As the enzyme is of plant origin the reaction does not need to be terminated with FBS. Following detachment, the cell suspension was transferred to a 15 mL Falcon tube, centrifuged (182× *g*, 5 min), decanted and resuspended in fresh medium. After determination of cell count and viability by trypan blue exclusion (minimus 90%), the cells were ready to use.

In the experiment, 1 × 10^4^ LMH cells were placed in each well of a collagen coated 96-well plate (BioCoat) and 100 μL of the complete culture medium was added to each well. The cells were incubated overnight at 37 °C in 5% CO_2_ to allow them to attach. The following day, settled dust extracts (0.5%) were diluted in Waymouth’s Medium with no FBS (1:4). The final concentrations of dust extract was 0.125%. The medium was aspirated from the 96-well culture and 200 μL of each tested sample was added to well in eight repeats. The control samples consisted of cells without dust extracts. The cells were incubated in a CO_2_ incubator at 37 °C with 5% CO_2_ for 48 h.

After incubation, the medium with tested dust extracts was gently aspirated from each well and 100 μL of MTT (0.5 mg/mL in PBS; Sigma-Aldrich, St. Louis, MO, USA,) was added and incubated at 37 °C in 5% CO_2_ for 3 h. Following incubation, MTT was carefully removed and formazan precipitates were solubilised with 50 μL of DMSO (Sigma-Aldrich, St. Louis, MO, USA). Absorbance was measured at 550 nm with a reference filter of 620 nm, using a TriStar^2^ LB 942 microplate reader (Berthold Technologies, Bad Wildbad, Germany). The absorbance of the control sample (untreated cells with dust extract) was taken to represent 100% cell viability. Cell viability (%) was calculated as follows: (sample OD (optical density)/control OD) × 100%; and cytotoxicity (%) as: 100-cell viability (%). Results were presented as means ± SD (standard deviation).

### 2.7. Volatile Odourous Compounds

Analysis of air quality included the measurement of the following volatile odorous compound concentrations: ammonia, methylamine, dimethylamine, trimethylamine, hydrogen sulfide, methyl mercaptan, ethyl mercaptan, propyl mercaptan, butyl carbon monoxide, carbon dioxide, oxygen, total organic carbon, acrolein, acetaldehyde, formaldehyde formic acid and acetic acid.

Air samples (0.02–0.06 m^3^) were collected in tedlar bags using an aspirator (EAS 1203; Emio, Wrocław, Poland). Analyses of the collected compounds were performed by GC/MS (Perkin Elmer Arnel 1115, Waltham, MA, USA and Bruker Daltonics Inc. 436-GC, Manning Park Billerica, MA USA) equipped with an RTX-1 column, PFPD, TCD and FID detectors, and using nitrogen and helium as carrier gases. The study was performed according to Polish standards (PN) (Table 3). Results were calculated for the conditions of 1013 hPa and 293 K.

**Table 3:** Detection standards.

No.	VOCs	Norm
1	acetaldehyde	ZBES/PB/17 eighth edition of 07.02.2012
2	acetic acid	ZBES/PB/16 seventh edition of 07.02.2012
3	acrolein	ZBES/PB/17 eighth edition of 07.02.2012
4	ammonia	PN-EN ISO 11732:2007
5	butyl mercaptan	PB GE 18 third edition of 16.02.2009
6	carbon dioxide	PB GE 22 (first edition of 03.02.2010)
7	carbon monoxide	
8	diethylamine	ZBES/PB/32 third edition of 02.01.2013
9	dimethylamine	
10	ethyl mercaptan	PB GE 18 third edition of 16.02.2009
11	ethylamine	ZBES/PB/32 third edition of 02.01.2013
12	formaldehyde	ZBES/PB/17 eighth edition of 07.02.2012
13	formic acid	PN-88/Z-04196.02
14	hydrogen sulfide	PB GE 18 third edition of 16.02.2009
15	methyl mercaptan	PB GE 18 third edition of 16.02.2009
16	methylamine	ZBES/PB/32 third edition of 02.01.2013
17	oxygen	PB GE 22 (first edition of 03.02.2010)
18	propyl mercaptan	PB GE 18 third edition of 16.02.2009
19	total organic carbon	PN-EN 13526:2005; PN-EN 12619:2002
20	triethylamine	ZBES/PB/32 third edition of 02.01.2013
21	trimethylamine	

Air samples were also collected in the scrubber at a flow rate of 0.6 m^3^/h. Ammonia and formic acid were determined using a spectrophotometer (CFA SAN spectrophotometer, SKALAR, Breda, Netherlands), according to the norms (Table 3). Total organic carbon was determined according to the norms (Table 3) using a portable, heated FID total hydrocarbon analyzer, OVF-3000 (J.U.M.^®^ Engineering GmbH, Karlsfeld, Germany).

### 2.8. Statistical Analysis

Statistical analyses was performed for number of selected microorganisms in settled dust as well as for share of granulometric fractions of dust and cytotoxicity properties of dust from different poultry farms. Statistical analyses were conducted using STATISTICA 10 software (Statsoft, Round Rock, TX, USA). Descriptive statistics for all variables of interest were calculated. The results were evaluated using one-way analysis of variance (ANOVA) at the significance level 0.05. When statistical difference was detected (*p* < 0.05), means were compared using Tukey’s post hoc procedure at the significance level 0.05.

## 3. Results and Discussion

In our study, the highest concentration of airborne dust was found in the PM_10_ dust fraction (average: 0.875 mg/m^3^, maximum: 2.128 mg/m^3^) in poultry farms. Dust particles with lower diameters PM_1_, PM_2.5_, PM_4_ were found at levels 0.480–0.541 mg/m^3^ (Table 4).

**Table 4:** Airborne dust concentration in poultry farm.

Dust Fraction	Concentration (mg/m^3^)
PM_1_	X: 0.480 ± 0.113
	Min: 0.201 ± 0.046
	Max: 1.080 ± 0.165
PM_2.5_	X: 0.493 ± 0.114
	Min: 0.206 ± 0.047
	Max: 1.101 ± 0.165
PM_4_	X: 0.541 ± 0.116
	Min: 0.227 ± 0.054
	Max: 1.189 ± 0.159
PM_10_	X: 0.875 ± 0.121
	Min: 0.352 ± 0.094
	Max: 2.128 ± 0.177
PM_total_	X:1.440 ± 0.132
	Min: 0.502 ± 0.143
	Max: 3.716 ± 0.431

PM_1_; PM_2.5_; PM_4_; PM_10_—respectively: granulometric fraction with diameter less than 1; 2.5; 4; 10 μm; X—mean; Min, Max—minimum and maximum value.

Ellen *et al.* [33] and Viegas *et al.* [34] carried out studies to determine dust concentrations in poultry farms. The authors found similar results, where PM_10_ predominated, albeit at higher concentrations (1.4–15.2 mg/m^3^). Concentration of airborne dust may depend on the type of bird (lying hens, broilers), the stage of the production cycle, the ventilation system in the poultry farm and other factors [33]. Granulometric fractions with particles of diameters <2.5 μm made up 1.0%–1.9% of settled dust (Table 5).

**Table 5:** Percentage share of granulometric fractions of settled dust.

Poultry Farm	Fractions of Settled Dust (%)		
	PM_2.5_	PM_10_	PM > 10
I	1.88 ± 0.26 ^B^	10.10 ± 1.04 ^B^	89.91 ± 1.04 ^D^
II	2.69 ± 0.09 ^A^	13.71 ± 0.42 ^A^	86.29 ± 0.42 ^E^
III	1.92 ± 0.32 ^B^	10.42 ± 1.07 ^B^	89.58 ± 1.07 ^D^
IV	1.81 ± 0.38 ^B,C^	10.17 ± 1.56 ^B^	89.83 ± 1.56 ^D^
V	1.43 ± 0.06 ^B,C,D^	9.84 ± 0.08 ^B,C^	90.17 ± 0.07 ^C,D^
VI	1.42 ± 0.01 ^B,C,D^	9.55 ± 0.10 ^B,C,D^	90.45 ± 0.10 ^B,C,D^
VII	1.14 ± 0.06 ^D^	7.37 ± 0.30 ^D,E^	92.63 ± 0.30 ^A,B^
VIII	1.27 ± 0.14 ^C,D^	7.69 ± 0.69 ^C,D,E^	92.31 ± 0.68 ^A,B,C^
IX	0.97 ± 0.08 ^D^	6.85 ± 0.35 ^E^	93.15 ± 0.35 ^A^
X	1.06 ± 0.16 ^D^	7.26 ± 0.63 ^E^	92.74 ± 0.63 ^A^
Mean	1.56 ± 0.52	9.30 ± 2.08	90.70 ± 2.08

PM_2.5_—granulometric fraction with diameter less than 2.5 µm; PM_10_—granulometric fraction with a diameter less than 10 µm; PM—granulometric fraction with a diameter more than 10 µm, ^A, B, C, D, E^—means with the same capital letter in the same column are not significantly different (Tukey’s test; *p* < 0.05).

Dust particles smaller than 10 μm made up 6.8 to 10.4% of the settled dust. Significantly higher (*p* < 0.05) percentages of both fractions (2.7% and 13.7%, respectively) were observed in farm II for PM_2.5_ and PM_10_. The percentage of particles with diameter more than 10 µm at farm II amounted to 86.3% and was significantly lower (*p* < 0.05) compared to others farms. However, most of the settled dust particles analysed were above 10 μm (89.6%–93.2%) (Table 5). This fact indicates the dominance of the same granulometric size fractions of dust in both the settled dust and the air. The dominance of this fraction is probably associated with a particular poultry farm’s specificity and dust composition—feathers, skin of birds, litter and feed particles falling from the air to the surface. Dust particles >10 μm are not capable of deep penetration into the human respiratory system, and are mostly deposited in the mouth and nose [35]. Exposure to PM_10_ may also produce disease by impacting the upper and larger airways below the vocal cord [34].

It is worth emphasizing that there are no regulations/limits for the percentage of each fraction in the settled dust in workplaces. Existing recommendations directly relate to concentrations of particulate matter in the air. According to the Approved Code of Practice (ACOP) the Control of Substances Hazardous to Health Regulations 2002 (COSHH) [36] any kind of dust can become hazardous to health when it is present at concentrations 10 mg/m^3^ or higher in the air (weighted arithmetic mean during eight-hour period) for inhalable dust, or 4 mg/m^3^ (weighted arithmetic mean during eight-hour period) for respirable dust. In our study, airborne dust did not exceed the recommended limits. It is worth emphasizing that the limits take into account single agent exposure. However in the tested working environments there exists co-exposure to several physical (including dust), chemical (odours) and biologic agents (pathogens).

The different groups of microorganisms in the settled dust of the poultry farms are shown in Table 6. Bacteria (average: 3.2 × 10^9^ cfu/g), and actinomycetes (5.5 × 10^6^ cfu/g) dominated all isolated microorganisms, while fungi occurred less frequently (1.2 × 10^6^ cfu/g). The total number of bacteria in settled dust samples varied depending on the poultry farms tested, and ranged from 1.5 × 10^7^ to 2.9 × 10^9^ cfu/g; significantly different (*p* < 0.05) bacteria number was detected in IV poultry farm 1.7 × 10^10^ cfu/g. The concentration of actinomycetes in tested dust samples was at a similar level, with the exception of farms I and IV which showed a statistically significant difference in their number (*p* < 0.05). In all tested dust samples, mannitol positive *Staphylococci* spp. (1.7 × 10^9^ cfu/g) and *Enterococcus* spp. (5.8 × 10^7^ cfu/g) were identified. *E. coli* was present in 90% of the analysed dust samples (with concentration 1.6 × 10^5^ cfu/g). *Pseudomonas fluorescens* was isolated from 4/10 dust samples (8.7 × 10^5^ cfu/g). A particularly high number of these bacteria was detected at poultry farm IV (*p* < 0.05). Only in 3/10 dust samples were *Salmonella* spp. identified (9.5 × 10^4^ cfu/g). A statistically significant number of bacteria belonging to *Salmonella* genera was detected on farm VIII (*p* < 0.05) (Table 6).

**Table 6:** Number of microorganisms in settled dust in poultry farms.

Poultry Farm	Microorganisms Number (cfu/g, Mean ± SD)							
	Total Number of Bacteria	Actinomycetes	Mannitol Positive *Staphylococci* sp.	*Enterococcus* spp.	*E. coli*	*Salmonella* spp.	*Pseudomonas fluorescens*	Fungi
I	2.8 × 10^9^ ± 1.2 × 10^9^ ^B,C^	1.4 × 10^7^ ± 7.8 × 10^6^ ^B^	2.6 × 10^9^ ± 1.4 × 10^9^ ^A,B,C^	1.2 × 10^8^ ± 4.2 × 10^7^ ^A^	nd	nd	5.3 × 10^4^ ± 8.0 × 10^3^ ^B^	1.8 × 10^5^ ± 4.6 × 10^4^ ^C^
II	2.9 × 10^9^ ± 1.5 × 10^9^ ^B,C^	3.4 × 10^5^ ± 6.1 × 10^4^ ^C^	4.4 × 10^9^ ± 1.9 × 10^9^ ^A,B^	1.6 × 10^7^ ± 5.0 × 10^6^ ^B^	9.7 × 10^3^ ± 5.1 × 10^3^ ^B^	nd	1.2 × 10^6^ ± 6.0 × 10^5^ ^B^	1.1 × 10^5^ ± 7.1 × 10^4^ ^C^
III	5.5 × 10^9^ ± 6.0 × 10^9^ ^B^	6.0 × 10^5^ ± 1.1 × 10^5^ ^C^	1.7 × 10^9^ ± 7.5 × 10^8^ ^B,C^	1.8 × 10^8^ ± 9.5 × 10^7^ ^A^	5.8 × 10^5^ ± 1.2 × 10^5^ ^A^	nd	nd	1.5 × 10^6^ ± 5.4 × 10^5^ ^B,C^
IV	1.7 × 10^10^ ± 4.5 × 10^9^ ^A^	7.7 × 10^4^ ± 4.5 × 10^4^ ^C^	5.1 × 10^9^ ± 2.0 × 10^9^ ^A^	1.4 × 10^8^ ± 1.2 × 10^7^ ^A^	2.1 × 10^4^ ± 9.6 × 10^3^ ^B^	nd	7.3 × 10^6^ ± 4.0 × 10^6^ ^A^	3.6 × 10^5^ ± 1.1 × 10^5^ ^C^
V	7.1 × 10^8^ ± 1.2 × 10^8^ ^C^	3.3 × 10^7^ ± 7.5 × 10^6^ ^A^	2.4 × 10^8^ ± 7.0 × 10^7^ ^C^	1.3 × 10^7^ ± 4.2 × 10^6^ ^B^	2.0 × 10^5^ ± 8.9 × 10^4^ ^B^	nd	nd	1.6 × 10^6^ ± 2.6 × 10^5^ ^A,B,C^
VI	4.4 × 10^8^ ± 7.0 × 10^7^ ^C^	2.4 × 10^6^ ± 7.0 × 10^5^ ^C^	6.2 × 10^7^ ± 1.2 × 10^7^ ^C^	9.7 × 10^6^ ± 4.0 × 10^6^ ^B^	2.5 × 10^5^ ± 8.0 × 10^4^ ^B^	nd	nd	5.6 × 10^5^ ± 2.7 × 10^5^ ^C^
VII	4.1 × 10^8^ ± 6.6 × 10^7^ ^C^	1.1 × 10^6^ ± 4.5 × 10^5^ ^C^	1.8 × 10^8^ ± 3.1 × 10^7^ ^C^	1.2 × 10^7^ ± 4.5 × 10^6^ ^B^	2.0 × 10^4^ ± 1.2 × 10^4^ ^B^	nd	nd	1.0 × 10^6^ ± 3.4 × 10^5^ ^B,C^
VIII	1.5 × 10^7^ ± 6.8 × 10^6^ ^C^	1.5 × 10^6^ ± 4.6 × 10^5^ ^C^	1.6 × 10^6^ ± 5.7 × 10^5^ ^C^	1.3 × 10^6^ ± 4.5 × 10^5^ ^B^	7.0 × 10^3^ ± 4.0 × 10^3^ ^B^	6.3 × 10^5^ ± 3.1 × 10^5^ ^A^	8.0 × 10^4^ ± 4.0 × 10^4^ ^B^	4.6 × 10^5^ ± 1.8 × 10^5^ ^C^
IX	2.0 × 10^9^ ± 4.2 × 10^8^ ^B,C^	1.2 × 10^6^ ± 2.1 × 10^5^ ^C^	1.6 × 10^9^ ± 5.7 × 10^8^ ^B,C^	8.0 × 10^7^ ± 3.0 × 10^7^ ^A,B^	5.3 × 10^5^ ± 2.3 × 10^5^ ^A^	1.1 × 10^5^ ± 6.0 × 10^4^ ^B^	nd	2.6 × 10^6^ ± 8.5 × 10^5^ ^A,B^
X	8.5 × 10^8^ ± 6.5 × 10^7^ ^C^	1.0 × 10^5^ ± 2.0 × 10^4^ ^C^	8.2 × 10^8^ ± 7.8 × 10^7^ ^C^	8.0 × 10^6^ ± 3.6 × 10^6^ ^B^	5.3 × 10^3^ ± 2.5 × 10^3^ ^B^	2.1 × 10^5^ ± 1.0 × 10^5^ ^B^	nd	3.2 × 10^6^ ± 1.9 × 10^6^ ^A^
**Mean ± SD**	**3.2 × 10^9^ ± 5.0 × 10^9^**	**5.5 × 10^6^ ± 1.1 × 10^7^**	**1.7 × 10^9^ ± 2.0 × 10^9^**	**5.8 × 10^7^ ± 7.0 × 10^7^**	**1.6 × 10^5^ ± 2.3 × 10^5^**	**9.5 × 10^4^ ± 2.1 × 10^5^**	**8.7 × 10^5^ ± 2.5 × 10^6^**	**1.2 × 10^6^ ± 1.1 × 10^6^**

SD—standard deviation; nd—not detected in 1 g of settled dust, ^A, B, C^—means with the same capital letter in the same column are not significantly different (Tukey’s test; *p* < 0.05).

Baker *et al.* [37] and Witkowska *et al.* [38] found that the number of aerobic bacteria in litter were at a level of 10^7^–10^9^ cfu/g; (from 9.5 × 10^7^ cfu/g, which increased to 2.3 × 10 ^9^ cfu/g after 5 weeks of broiler settlement). We found similar bacterial genera in settled dust in our study as described in other studies on poultry farms in literature: *Bacillus*, *Clostridia*, *Corynebacterium*, *Enterobacter*, *Flavobacterium*, *Pseudomonas*, *Staphylococcus*, *E. coli*. [39].

According to Directive 2000/54/EC [5] *Enterococcus*, *Escherichia coli* and *Salmonella* that were isolated in our study, can be harmful to workers. Dutkiewicz *et al.* [6] also indicated that *P. fluorescens* is potentially dangerous to people with an impaired immune system. Moreover, they also found the following potentially pathogenic bacteria in study: *Chlamydia ornithosis*, *Bacillus anthracis*, *Salmonella* Choleraesuis var. Typhi, *Listeria monocytogenes*, *Mycoplasma* spp., *Staphylococcus aureus*, *Streptococcus* spp. which can occur in manure, litter, settled dust. We also isolated 13 strains of fungi in settled dust, which included three strains of yeast and 11 mould strains (Table 7).

**Table 7:** Fungal species isolated from settled dust samples.

No	Species	Frequency of Isolation (%)	Percentage of Isolation (%)	Isolation Place (Poultry Farm)
1	*Absidia glauca*	27.5%	7.2%	I–III; V; VII; X
2	*Alternaria alternata*	10.0%	0.8%	III; VI
3	*Aspergillus fumigatus*	15.0%	5.3%	V; VI
4	*Aspergillus penicillioides*	72.5%	32.5%	I–X
5	*Candida pelliculosa* ^A^	15.0%	2.3%	III; VII; VIII
6	*Cephaliophora tropica*	5.0%	0.0%	II
7	*Chaetomium globosum*	15.0%	0.7%	II; VI; X
8	*Cryptococcus uniguttulatus* ^B^	25.0%	8.5%	I–VI
9	*Eurotium chevelierii*	32.5%	15.2%	IV–V; IX; X
10	*Mucor fragilis*	27.5%	20.2%	II; IV; V; VII; VIII; X
11	*Mucor pirimiformis*	17.5%	2.1%	I; II; V; VI; VIII
12	*Paeciliomyces variotii*	60.0%	4.0%	I–X
13	*Rhodotorula mucilaginosa* ^C^	27.5%	1.2%	I–V; VIII

^A, B, C^—compatibility of the identification of yeasts obtained by APIweb system respectively: 99,8%; 99,9%; 99,9%.

The following strains were isolated at the highest frequencies: *Aspergillus penicillioides* (72.5%), *Eurotium chevelierii* (32.5%), *Mucor fragilis* (27.5%) *Absidia glauca* (27.5%). These species also had the highest percentage of isolations (quantitatively determined) in dust samples, ranging between 5.3% and 29.1% among the isolated colonies (Table 7). Mould species isolated from settled dust samples were saprophytic organisms and are specific to environments such as soil or plant material [28]. According to the literature, the most common fungal genera reported in settled dust include *Cladosporium*, *Penicillium*, *Aspergillus*, although *Alternaria*, *Fusarium* and *Geotrichum* can also be present [39]. *A. fumigatus*, one of the moulds identified in settled dust samples in two poultry farms, belongs to the second health risk group according to Directive 2000/54/EC [5], which concerns protecting workers from risks related to biological agents in work places. This species is known for its infection ability, allergenic and toxinogenic properties [27]. Also, *Paecilomyces variotii*, a species with a relatively pronounced ability to survive in vertebrate tissue, is classified to BSL2 (biosafety level 2) by the European Confederation of Medical Mycology (ECMM) [40]. In severely immunocompromised people, it may cause serious opportunistic mycoses [40]. The literature also identifies other potentially patogenic species from poultry farm environments: *Candida albicans*, *Cryptococcus neoformans*, which we did not find in our study. However, we identified another species from the genera *Candida* (*C. pelliculosa*) and *Cryptococcus* (*C. uniguttulatus*).

In the settled dust samples, 27 chemicals classified as secondary mould metabolites were identified (Table 8). Metabolites identified in high concentrations were: aurofusarin, deoxynivalenol, 15-hydroxyculmorin zearalenone, zearalenone-sulfate (VIII), infectopyron (VIII–X), and neochinulin A (IX, X). Asperglaucide, brevianamid F, enniatin B, enniatin B1 were detected in all samples. Other compounds varied by the poultry farm where the dust sample was isolated.

The highest numbers of secondary metabolites were detected in the dust sample isolated from poultry farm VIII. It included typical *Fusarium* spp. mycotoxins: 15-Hydroxyculmorin apicidin, aurofusarin, α-zearalenol, β-zearalenol, deoxynivalenol, *epi*-equisetin, equisetin, zearalenone and zearalenone-sulfate. Among the moulds isolated, the genus *Fusarium* was not detected. This may be due to previously contaminated plant material e.g., feed for poultry.

Dust from poultry farms contains low levels of mycotoxins (e.g., trichothecene B., deoxynivalenol, zearalenone) [41]. Literature data confirms that secondary metabolites of the *Fusarium* genus are a common feed contamination. The most common mycotoxins found in grains for poultry feed are deoxynivalenol (DON) and its derivatives. [42]. In the studies presented, DON was identified at a poultry farm (VIII) at a concentration of 151.2 mg/kg dust. High exposure to DON can lead to nutrition disorders, metabolism and immunity [43]. In all poultry farms, Zearalenone (ZEN) was detected at 0.7–72.2 mg/kg dust. ZEN derivatives were also detected on farms VII and VIII–X: zearalenone-sulfate (42.7–204. 5 µg/kg) and α-zearalenol (36.9 µg/kg). It is worth noting that ZEN has relatively low toxicity, with an LD_50_ value of 2 ± 10 g/kg body weight determined for mice [44]. Its role as a mammalian endocrine disrupter and its genotoxicity potential was previously reported [45].

**Table 8:** Secondary metabolites in settled dust samples.

Secondary Metabolite	Concentration (μg/kg)									
	I	II	III	IV	V	VI	VII	VIII	IX	X
15-Hydroxyculmorin	-	-	-	-	-	-	-	114.58	-	-
3-Nitropropionic acid	1.69	3.42	1.64	1.67	2.70	-	2.48	-	-	-
α-Zearalenol	-	-	-	-	-	-	-	36.93	-	-
Alternariol	-	-	-	-	-	-	-	2.42	2.53	-
Altersetin	-	-	-	-	-	-	-	11.51	13.36	9.83
Apicidin								3.18	-	2.87
Asperglaucide	5.21	38.26	0.39	0.05	32.68	31.30	13.39	45.23	46.24	51.09
Aurofusarin	2.96	3.56	-	10.46	15.07	9.79	14.96	281.44	-	-
Beauvericin	-	-	-	-	-	-	-	1.25	1.94	-
β-Zearalenol	-	-	-	-	-	-	-	8.14	-	-
Brevianamid F	3.56	3.06	1.26	1.99	3.58	2.89	1.18	63.15	32.67	65.95
Citreorosein	-	-	-	-	-	-	-	-	13.18	-
Deoxynivalenol	-	-	-	-	-	-	-	151.20	-	-
DON-3-glucoside	2.91	-	-	-	-	2.22	3.76	-	-	-
Emodin	-	-	0.22	-	0.28	0.26	0.41	7.15	17.41	2.88
Enniatin B	0.09	0.12	0.15	0.13	0.31	0.95	0.12	2.13	0.67	0.70
Enniatin B1	0.09	0.12	0.11	0.11	0.28	1.30	0.12	1.96	0.94	0.95
Equisetin	-	-	-	-	-	-	-	1.92	1.84	2.37
Infectopyron	-	-	-	-	15.87	21.52	7.62	249.12	125.49	122.26
Mycophenolic acid	0.26	4.13	-	-	-	-	-	-	-	-
Neoechinulin A	1.73	4.26	0.14	-	0.94	0.26	0.65	22.93	426.40	784.48
Nivalenol	-	-	-	-	-	-	-	-	19.57	24.88
Siccanol	6.37	18.42	11.17	11.45	9.36	8.26	6.13	-	-	-
Zearalenone	1.62	0.71	1.16	1.32	3.04	2.07	7.30	72.18	-	-
Zearalenone-sulfate	-	-	-	-	-	-	-	204.48	42.72	49.52

“-“—not detected.

In this study, characteristic metabolites of the following genera were detected in settled dust: *Alternaria* (alternariol, altersetin, infectopyrone), *Aspergillus* (3-nitropiopropionic acid), *Penicillium* (mycophenolic acid). Also nonspecific metabolites—whose occurrence cannot be linked to specific moulds (asperglaucide, brevianamid F, citreorosein, emodin, enniatin B, enniatin B1, neoechinulin A) were identified in dust samples. Mould secondary metabolites can be a contamination of the litter, and feed coming from other sources (e.g., walls with mould growth on premises for poultry breeding that with air movements can get into the dust. Secondary metabolites are stable in the environment and they can be detected after mould death.

**Figure 1:**
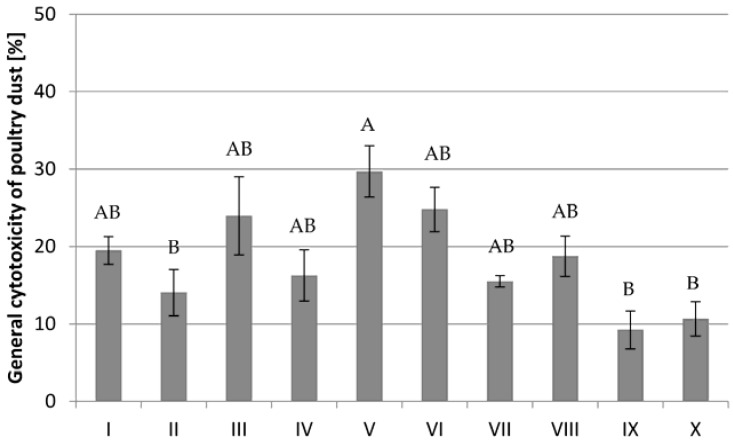
Cytotoxicity of poultry dust measured in MTT assay against LMH chicken cell line after 48 h exposition. Each point represents the mean of eight individual well absorbance values (± SD) for I–X poultry farm. A, B—means with the same capital letter in the same column are not significantly different (Tukey’s test; *p* < 0.05).

Settled dust cytotoxicity was tested on chicken hepatocyte cells, LMH (Figure 1). Tested dust samples showed weak cytotoxicity in the range of 9.2%–29.7%. Only in the case of three samples (III, V, VI), the cytotoxicity of dust towards chicken hepatocytes was higher than 20%, but still it was not significantly different (*p* < 0.05) from other farm samples.

We can hypothesize that in poultry farms, metabolites such as mycotoxins and endotoxins may be present in the form of dust suspended in water vapour, and can be inhaled by birds and people. According to our results, settled dust in poultry farms, is not a toxic agent for chicken hepatocytes under evaluated conditions. There is very little research on the influence of settled dust on birds and human health.

It was also documented that volatile odorous compounds are attached to dust particles [46]. The volatile odorous compound concentrations in the air measured in poultry farms during a production cycle is presented in Table 9.

Oxygen and carbon dioxide was present at the highest concentration in all tested farms. Among volatile odorous compounds ammonia, acrolein, methyloamine, acetic acid, acetoaldehyde, and formaldehyde, were detected. We observed a variability in the concentrations of individual compounds depending on the farm and stage of the production cycle in farm II.

Analyses of odorous volatile compounds in poultry farm II were conducted before the production cycle started, and on the 20th and 35th day of breeding. Comparison of volatile odorous compound concentrations indicated that ammonia, carbon dioxide, acetaldehyde and acetic acid concentrations increased during broiler breeding cycles.

The amounts of ammonia on farms XI and XII were high at the end of the cycle: 16.8–66.7 mg/m^3^. The limit according to Council Directive 2007/43/EC is 20 ppm, ~14 mg/m^3^ under conditions of 1013 hPa and 293 K [23]. According to the requirements of the Council Directive 2007/43/EC [23], the maximum concentration of ammonia did not exceed permissible limits only at farm II, but the concentration of carbon dioxide exceeded the admissible value of 3000 ppm (~5490 mg/m^3^ under conditions of 1013 hPa and 293 K) in the third stage of the production cycle at poultry farm II. At farm XI the concentration of carbon dioxide was very close to the limit. Assessing the impact of the substances analysed on the quality of perceived smell from poultry operations, we noted that only the concentration of acetic acid (10.02 mg/m^3^) at the poultry farms (stage II) clearly exceeded the olfactory threshold (2.0 mg/m^3^) [47]. However, trimethylamine, mercaptans and hydrogen sulfide were detected in small concentrations. Hence, it can be expected that the sensory perception of smell is also influenced by these substances.

**Table 9:** Concentration of volatile odorous compounds in the air.

No.	Compounds	Concentration (mg/m^3^)				
		Farm XI	Farm XII	Farm II during Production Cycle		
				I Stage	II Stage	III Stage
1	Acetaldehyde	0.00449	0.00691	0.00437	0.00636	0.00760
2	Acetic acid	<0.11	<0.11	<0.11	10.02	0.15
3	Acrolein	<0.017	0.135	0.145	<0.017	<0.017
4	Ammonia	16.85	66.7	0.81	2.12	1.59
5	Butyl mercaptan	<0.42	<0.41	<0.43	<0.41	<0.41
6	Carbon dioxide	5464	4459	1039	4704	6564
7	Carbon monoxide	<129	<129	<132	<129	<0.13
8	Diethylamine	<0.28	<0.28	<0.29	<0.28	<0.28
9	Dimethylamine	<0.22	<0.22	<0.22	<0.22	<0.22
10	Ethyl mercaptan	<0.27	<0.29	<0.29	<0.28	<0.28
11	Ethylamine	<0.36	<0.36	<3.70	<0.36	<0.36
12	Formaldehyde	0.07067	0.07577	0.08544	0.09168	0.06900
13	Formic acid	<0.0015	<0.002	<0.002	<0.0015	<0.0015
14	Hydrogen sulfide	<0.16	<0.16	<0.16	<0.16	0.10
15	Methyl mercaptan	<0.22	<0.22	<0.23	<0.22	<0.22
16	Methylamine	<0.66	<0.66	<0.68	0.82	<0.66
17	Oxygen	286,147	305,166	316,512	304,625	249,871
18	Propyl mercaptan	<0.35	<0.35	<0.36	<0.35	<0.35
19	total organic carbon	<1.60	<1.6	<1.60	<1.60	<1.60
20	triethylamine	<0.22	<0.22	<0.22	<0.22	<0.22
21	trimethylamine	<0.13	<0.13	<0.13	<0.13	<0.13

Stages: I stage—(without chickens); II stage (20th day), III stage (35th day), XI and XII (57th week, at the end of the cycle production).

Data on threshold values concerns single chemical constituents without other chemicals present in the air. Very limited data are available on the simultaneous, combined effect of different odorous substances on the overall odour. Such an effect, caused by the mixture of substances in the exhaust air produced from poultry units, may be the source of the unpleasant smell, even though single odorants are emitted only in very small amounts.

## 4. Conclusions

Settled dust can be a carrier of microorganisms, odours and secondary metabolites in poultry farms. Airborne total dust concentration on poultry farms was 1.44 mg/m^3^ with a high percentage of the PM_10_ fraction. Microorganisms were detected in settled dust at the following levels: 3.2 × 10^9^ cfu/g for bacteria and 1.2 × 10^6^ cfu/g for fungi. Potential pathogens (*Enterococcus* spp., *Escherichia coli*, *Salmonella* spp., *Aspergillus fumigatus, Paecilomyces variotii*) were also found in the settled dust. Secondary metabolites detected include aurofusarin, deoxynivalenol, 15-hydroxyculmorin zearalenone, zearalenone-sulfate, infectopyron, and naochinulin A, but tested dust samples showed weak cytotoxicity towards chicken hepatocyte cells in the range of 9.2%–29.7%. Among volatile odorous compounds ammonia, acrolein, methyloamine, acetic acid, acetoaldehyde and formaldehyde were detected in the air of the tested poultry farms.

Settled dust, airborne microorganisms, mould secondary metabolites and odours detected in poultry farms working environment may result potential respiratory health implications for workers. Future research should focus on medical respiratory tests of workers taking into account all factors constituted occupational risk (physical, biological and chemical) in poultry farm working environment. Also threat prevention methods e.g., assessment of the effectiveness of Filtering Respiratory Protective Devices dedicated to poultry farm staff should be developed. Moreover, studies should be undertaken to test the effectiveness of innovative systems for the purification of air and dust, and eliminating odour from poultry farms.

## Abbreviations

The following abbreviations are used in this manuscript:

DON: Deoxynivalenol
LC-MS/MS: Liquid Chromatography–Mass Spectrometry
LMH: Leghorn Male Hepatoma
MTT: 3-(4,5-dimethylthiazolyl-2)-2,5-diphenyltetrazolium bromide)
OD: Optical Density
PM: Particulate Matter
VOCs: Volatile Odorous Compounds
ZEN: Zearalenone
